# Bombay Report on Epidemic Cholera

**Published:** 1832-07-01

**Authors:** 


Reports on the Epidemic Cholera which has raged throughout Hindostan and the Peninsula of India, since August, 1817. Published under the authority of Government. 8vo. pp. 214, with Appendix, pp. 14. Bombay, 1819.

The present is the Report on the Epidemic Cholera drawn up with the concurrence of the Bombay Government, and published at its expense. It is what is usually known as the Bombay Report, and presents, when conjoined with those from Madras and Bengal, a complete and most valuable history of the epidemic cholera, as it shewed itself in India. Before public attention was earnestly and anxiously directed to every source of information respecting this singular disease, the labours of the Indian Boards were comparatively little regarded. The danger seemed too remote to excite attention or to warrant alarm, and although a few physicians, attracted by curiosity or by partial motives, devoted a slight degree of consideration to the subject, it was never fully investigated even by them. Many bad consequences have ensued from this general professional ignorance, but the effects resulting from the state of imperfect information and semi-knowledge, disseminated by the dabblers in the Indian reports, have been infinitely worse. Statements of false facts have been boldly made, and pertinaciously persisted in; a colouring has been given to other facts and opinions which possessed a real existence; general impressions have gone abroad founded rather on such bases as legendary tales, than sober historic records; and the result has been error, uncertainty, and confusion. It is to clear these mists and dispel these delusions, that we have undertaken to supply a full and analytical account of the several reports to which we have alluded. We trust that when we have accomplished our task we shall cease to hear and read those clashing statements of what was thought and done in India, which now incessantly distract the public. The profession will possess through the medium of this Journal, the means of comparison, the first accurate data on which to found conclusions respecting the similarity or dissimilarity of the cholera in India, Russia, England. It is idle, it is worse than idle, to waste words in disputation. We have had enough, and more than enough, of this and of assertion; we trust that hereafter we shall find a greater semblance of rigorous deductions from acknowledged facts.

The epidemic which is now upon us, which has invaded others before us, and may attack many hereafter, is certainly anomalous in its characters. Yet we do not ourselves see why the inductive mode of reasoning should not be as applicable to this as to any other malady, nor do we yet forego all hope that because the world has been hitherto unsuccessful in the inquiry, it must necessarily remain so. If men will perversely bar their eyes against all light, or admit it only through one green glass of theory, prejudice, credulity, or subserviency to authority, then the prospects for truth are hopeless indeed. But this is not the inductive method, nor any other that we know of, excepting that of the sheep of Panurgus, who jumped into the sea because their leader fell in. If men will cry out, before they so much as gain a glimpse of one patient, that cholera is contagious, or absolutely non-contagious, and, if after seeing it they twist all facts to their preconceived opinions, credulously admitting any thing on one side, obstinately denying every thing upon the other, those men are not calculated to advance science or benefit mankind; they are the remnants of the bigots and persecutors of all ages, the mark of the beast is upon them, they are not philosophers but fools, absolutely and unconditionally fools.

The cry, the stupid cry of contagion, in the sense in which it was employed, and the mode in which it was concocted, would have proved, if successful, a curse to the community, a blight to science, a barrier to truth, and a damning stain upon the century. Fortunately it was as futile as foolish. Our minds are yet left open to the impression of facts, and truth may come, without the certainty of the door being slammed in her face, unless she wear the badge and livery of a creed.

There is no more certain mark of a weak mind than prejudice, of a bad mind than virulent party animosity, of a mind at once malignant and petty than the mixture of both. That mixture of weakness and wickedness has been most apparent in the mode of conducting the discussions on the present epidemic. A party of men, determined from the consideration of a certain sort of evidence, to consider the disease highly contagious, were equally determined to hold up all of a contrary opinion as fools or knaves. These modest bigots wondered at the construction of their opponents' minds, just as an Indian may wonder at the constitution of mechanism, which surpasses the tomahawk or fishing-tackle of his own rude hut. It is indeed astonishing with what tenacity poor and sterile minds will cling to opinions hastily embraced. They seem to be much in the situation of a young couple, who having made a love match against all prudential and rational considerations, adore each other with such fury that nothing short of death can effect their separation. It certainly has been thus with the contagionists, and they and their theory were wedded and bedded, in despite of all prayers for reasonable deliberation and delay.

It is the fashion to consider the present age an intellectual one, and possibly it is so. But in proportion as the many display liberality and a disposition to woo and win true knowledge, to seek her as she should be sought, and to use her as she should be used, the few appear anxious to distinguish themselves by an opposite line of conduct. We know but one mode of arriving at truth, and that is by a careful study of facts, and cautious induction from them. We know but one mode of ascertaining facts or of verifying them, and that is by the employment of the eye of sense under the superintendence of the eye of reason. But in this most intellectual nation of a self-styled intellectual age, such a method of study is unblushingly scouted. Our senses are not to be employed, our opinions are to be bought or borrowed second hand from this or that hebdomadal journal. The evidence on which we are to found the most important conclusions is to be drawn from abroad, nay even from particular sources abroad, and the dicta of others are not to be confirmed or confuted by what we ourselves may see. A set of men, some two or twenty, constituted a body, are to decide on what they have never witnessed, are to lay down the law to those who have the means of judging for themselves, and that law is to be received with more submission than any edict of despotism, is to be viewed as unalterable as the ordinances of the Persians and the Medes. The law being laid down we are thenceforth to become mere passive instruments, automata to move where the wires are pulled. We are not to investigate the phenomena of a malady before us because investigation is a sin, a crime against the majesty of boards, juntas, and journalists. When the disease is in other lands, we are then, and then only, to say what it is, whence it comes, and whither it goes. When it springs up in our own, a change is to come on us. We must hie us from the public places—we must shun all contact with the malady—we must not view it, 'twould be sacrilege; we must not think of it, 'twould be treason; we must not talk of it, 'twould be quackery. We must get us to our closets, and thence we may pen pamphlets or write articles on a disease, which we can only know because we have not seen it. Such has been the impudent and stupid attempt to fetter opinion in this country. That it should miscarry so signally as it has done, was probably not contemplated by those who ventured to essay it. The result might, indeed, have been anticipated by those who can decipher the signs of the times, the impatience of authority not morally just, the prevalence of a spirit of liberality and freedom, mental and political, the diffusion of information, and the enthusiasm of men not chilled by the cold fetters of formal associations. The issue of this very dispute on the contagion of cholera, is to us no mean indication, amidst the many, of the mighty revolution that is yet but begun in the sentiments and manners of men.

To return to our immediate subject. The temper in which we would have men investigate this, as other questions of medical doctrine or medical polity, should be one of strict impartiality. Let them first procure facts, as far as such can be depended on; and where *they* fail, let them not, we implore them, fill up the void with prejudice and party-spirit. Let them not, as too many have done, and the bigoted few yet persist in doing, refuse the evidence of their own senses, and receive in preference the bias of theory and the dictates of authority. We repeat that it is to promote this desirable object, by diffusing genuine information and evidence which may safely be relied on, that we have determined to lay before the public the substance of the reports of the medical boards of Indostan. We shall give it in as terse a manner and concise a form as circumstances will permit.

The Bombay Report is drawn up by Messrs. R. Steuart and B. Phillips. It contains a preface of forty-two pages, embodying the conclusions formed by these gentlemen from the reports presented to them, as well as, we presume, from the evidence of their own senses. The remainder of the work is occupied by forty reports, from surgeons who had charge of detachments or hospitals in which the disease broke out. We shall arrange the prominent features of these individual communications in a tabular manner, as we did, in our last Number, with those published by the Madras Board.

The Bombay reporters commence by an extract from Mr. Jukes' letter, describing the first appearance of the disease. When it shewed itself at Jaulnah, it was pretty clear that it would reach Bombay. Passing over a space of 200 or 220 miles, visiting in its course Aurungabad and Ahmednuggur, it reached Seroor, a distance of 150 miles, on the 18th or 19th of July. Towards the latter end of the same month, it appeared in the city of Poonah, though the troops encamped in the neighbourhood remained healthy for some time afterwards.

"On the 6th of August it broke out with great violence at Panwell, a considerable village on the main line of communication between Poona and Bombay, separated from the latter by an arm of the sea, and distant about 15 or 20 miles; but between which a pretty constant communication is kept up by means of boats. On 9th or 10th of the same month the first case appeared on this Island, and, as appears by Doctor Taylor's report, could be traced to a man who had arrived from Panwell the same day; it is also evident by Mr. Jukes's report, that it spread north and south along the sea-coast from the same place, and that it was imported to a village in the neighbourhood of Tannah on the island of Salsett, distant from this place about 20 miles, by a detachment of troops that escorted a state prisoner to that garrison from Panwell. The disease did not break out at Mahim, on the extremity of this island, distant only 5 or 6 miles from the principal native town of Bombay, until it had been established in the latter: it then gradually spread over the western side of the island of Salsett, through which the road from Bombay to Surat and the northern countries lies, and by which, during the south-west monsoon, is the principal line of communication. By the observation of some individuals, who, aware of the danger of the malady, and with the humane view of relieving the sufferings which it inevitably produced, carefully watched its progress, we are enabled to trace the disease as if creeping along from village to village on that island precisely in the same way, that is, by the arrival of people affected with the disease from places where it was known to prevail; and we are assured that there are some small villages on that island, which from want of this sort of communication, *or from some other cause*, have, after a lapse of four months, hitherto escaped entirely." xi.

The Board conclude that the epidemic cholera differs from all other epidemics, and may be said to stand alone. It prevailed in temperatures varying from 40° Fahr. to 90 or 100, during incessant rain, and that arid state of atmosphere that leaves not a trace of vegetation. The Board also think the disease "capable of being transported from one place to another, as in cases of ordinary infection or contagion, and that it possesses the power of propagating itself by the same means that acknowledged contagions do, subject, however, to *particular* laws, with which we may never become acquainted." This exception rather eats than proves the rule. The Board are "aware of the doubtful nature of the ground on which they tread," and, therefore, mention some facts, with the view, we suppose, of supporting their opinions.

"In October last, when the disease had almost disappeared at Tannah, the attention of Mr. Jukes was called to a case that had appeared in one of the apartments of the barracks of that fort, appropriated to European troops; this, owing to too late application for medical aid, soon terminated fatally; another case occurred a few hours afterwards, the subject of which was saved with much difficulty and danger, and in the course of 6 succeeding days, no less than 9 cases occurred *in the same apartment*. The curiosity of Mr. Jukes was naturally excited, to ascertain under what circumstances so much disease was produced, and on examination, the ward appeared to be both badly ventilated and too much crowded with men, the place was immediately emptied, scoured, and fumigated, *after which no other case occurred.* Since the middle of December, when we had flattered ourselves that the disease was vanishing, as the cold season advanced, the number of cases considerably encreased in this Island, Salsett and the Conkan, and consequently excited much alarm; in some instances these cases have been confined to particular spots, and sometimes to particular houses, where the disease has attacked and destroyed in succession whole families, consisting of three, four, and five persons, while in others only a single case, or at most very few have occurred. We are utterly ignorant of any local circumstances to which such a change can be ascribed, unless by supposing that a diminution of temperature, together with exposure, may have called into action some latent remains of an active poison; otherwise it seems difficult to reconcile those facts with what it is observed in ordinary epidemics.

It will be observed that Mr. Jukes, in his report, remarks that the disease as it first appeared at Tannah, did not go through families when one had become affected; he has since seen sufficient reason to alter his opinion in regard to that particular; and we think that we have observed in several instances, that the disease has shewn a greater tendency to spread, where the first attacks have proceeded in their course to a fatal termination, which they invariably do when not counteracted by medicine. How far the same thing has been observed to happen in other epidemics we cannot determine." xiv.

For a description of the disease and of its symptoms, the reporter quotes the words of a letter from the Bengal Board: these we need not insert here. In that letter the occurrence of consecutive fever is mentioned, but in a manner which is far from applicable to its character in the epidemic cholera of Europe. The Bombay Board, however, distinctly deny its existence on that side of India, and although that denial has been previously alluded to in this Journal, we cannot refrain from explicitly inserting it in this place.

"After the above luminous description, and what will be found in the reports which follow, it appears quite superfluous to enter here into any farther detail of the symptoms of this disease; we shall only therefore mention, that the subsequent fever which it appears has generally accompanied it in Bengal, has been but little if at all observed on this side of India; and as we have before noticed may be owing in a great degree to the more extended influence of those causes which are known to produce the bilious remittent fever as an epidemic in the Bengal provinces than on this side of the Peninsula; for it can scarcely be supposed that a disease, so uniform in its attack and in its course, should, as it were, deviate from itself in any considerable degree, without the agency of some local cause; and we entirely agree in the opinion that it cannot be fairly considered as forming a part of it." xxii.

The Board go on to notice the accurate description of the disease which Sydenham has given us. The logical consequences of this observation were pointed out in our last number, and need not again be insisted on. After noticing the fact of cholera having been described by other authors, and by Dr. James Johnson the latest, and after endeavouring to offer an etymological explanation of the term, *mort-de-chien,* and *mordexim,* the reporters make the following remarks on the question of contagion.

"The exciting and proximate causes of this interesting epidemic, although of the greatest importance to be understood, are like those of most other epidemical diseases, concealed under complete obscurity, '*atra caligine mersæ.*' Great difference of opinion exists among practitioners, as to its contagious or non-contagious influence, and this difference very naturally arises out of the difficulty of the subject; and when we consider the various and opposite opinions entertained by the most experienced practitioners of Europe on the same question, respecting the influenza of 1803, and the divided sentiments which have so long agitated the medical world on the subject of the yellow fever, and even typhus itself, we do not venture at present to decide on so important a point. Several irresistible facts already noticed or related in the following reports and its marked anomaly from all hitherto known simple epidemics, would seem to favor the doctrine of contagion, while the contrary supposition is only supported by a species of negative evidence. This is a question however of the greatest importance and ought not to be too hastily entertained as proved, nor rejected as unfounded; but prosecuted with that diligent enquiry and cautious induction which on every subject of science are so necessary to the attainment of truth; and we entertain a confident hope that the wide range through India, which the disease has taken, will have afforded to some gentlemen more ample means of determining it than we possess." xxx.

The predisposing causes are more obvious, and on this point more unanimity of opinion has prevailed.

"Rapid atmospherical vicissitudes in regard either to temperature or moisture; exposure of the body to currents of cold air, particularly the chill of the evening, after being heated by violent exercise of any kind inducing debility or exhaustion; low marshy situations; insufficient clothing; flatulent and indigestible food, especially crude and watery vegetables, which compose a large proportion of the diet of the natives; and particularly that gradual undermining of the constitution which arises in a condensed, dirty, and ill-fed mass of population, are all unquestionably powerful predisposing causes; and though not necessary to the production of the disease, do, when present, offer a more unlimited range to the operation of the original cause, whatever that may be. Sad experience has however shown that the absence of all those affords no security against the attack; although it appears that a much smaller proportion of the higher orders of society have suffered from it on this side of India, than in the Bengal provinces; *and in this Island, the disease has been confined almost exclusively to that class who are most exposed to the severest labour and privation.*" xxxi.

Here we see a great feature of similarity between the Indian and European epidemic; we allude to its choice of low and paludal localities, and of the poor and the wretched as its victims. When we say that the speculations of the Board on the proximate cause of the disease are unsatisfactory, we accuse them of no more, than all who have ventured to theorize on the subject have been guilty of. They are inclined to think that sometimes the poison acts more immediately on the vascular, sometimes on the nervous system. The Board make no mention whatever of premonitory symptoms, in fact they explicitly state that the first perceptible ones consist in a spasmodic affection of the stomach.

"The most general attack seems to consist in a spasmodic affection of the stomach, duodenum, and more especially the biliary ducts (the total absence of bile in the matter voided upwards or downwards being perhaps the most uniform characteristic of the disease,) which quickly extending through the whole intestinal canal, discharges its contents; for it has often been observed that the purging more resembles the forcible squirting from a syringe, than the operation of a common cathartic. It is more than probable, however, that these are merely the first perceptible symptoms, for it would appear that a great change has already taken place in the circulating system, and that the action of the heart itself has been greatly diminished before they occur. This seems evident from the numerous cases in which neither vomiting nor purging are present, and in which the first appearance of disease is the almost total suspension of the vital functions, immediately followed by severe spasmodic affections of the muscles, and coldness of the extremities. It is said that a diminution of the nervous influence occasions contractions or spasms of the muscles, and it is perhaps equally probable that a diminution of the stimulus of the circulating fluid, and especially of the vital heat which it constantly supplies, may produce the same effect. This, indeed, appears to lay the foundation of the cold stage, and the chain of distressing symptoms that accompany it. There are perhaps few diseases attended with such fatal effects, to which the human frame is subjected, of which so little of the first attack has been observed by practitioners; and this may be perhaps easily accounted for by the insidious nature of the attack itself, which is generally unaccompanied with any alarming symptoms, but more particularly when we consider the nature and circumstances of those subjects who have been chiefly presented to our view. They are composed of the poor and labouring classes, who are occupied in obtaining subsistence for the day that is passing, and who, while the excitement which labour and exercise produce remains, may feel but little inconvenience; but the moment that ceases, may speedily become its victims; hence it has been generally observed that the attacks are most frequent in the night." xxxiv.

The Board moot the question, whether the disease is ever ushered in among the natives by a stage of excitement. Dr. Burrell asserts, in his report, that among Europeans it decidedly is so, unless prevented by particular circumstances, as exposure to a draught of cold air.

"As far, too, as we have had opportunities of observing the commencement of the disease in natives (which have been confined chiefly to domestic servants, who, fully aware of its fatal nature, seemed anxious to avail themselves of the remedies which they knew we were ready to administer, and which may have amounted to twenty or thirty cases), after a few watery stools, or pain in the bowels, sometimes accompanied with vomiting, we could distinctly perceive a preternatural heat of the skin, with a small, quick and thready pulse, laborious breathing, and in some attended with such a change of features and countenance as to render them with difficulty recognizable by their employers. In such cases, if medicine be not immediately exhibited, it is equally certain that the disease, in a few hours, assumes all the worst forms that have been described, namely, coldness, sinking of the pulse, spasms, and death. It must, however, be acknowledged, that this stage of excitement is by no means so distinctly unfolded among the natives as among Europeans, and this may depend upon constitutional causes." xxxvi.

The Board here draw attention to the description of congestive typhus given by the late Dr. Armstrong, and observe, that those who are most intimate with the various forms of the attack of cholera, will be struck with the similarity of the two diseases *at their first appearance*, while experience has proved that they are best met by the same remedies.

Lastly, the Board advert to the subject of treatment. On this we may be brief. Blood-letting they consider the sheet anchor *in Europeans*. Now we know that the great majority of Europeans in India are persons in the prime of life, troops, for instance, and in comparative vigour. On the contrary, we have the authority of the Board for the fact, that those attacked among the natives were, in Bengal, as here, the poor, squalid, and debilitated. With them blood-letting lost its powers, by the same rule and for the same reason, that blood-letting was found even mischievous amidst the half-starved Irish in Southwark. Next to blood-letting the Board rank calomel, which often preserved life when the former could not be put in practice. When the disease was met on its first attack, a single scruple dose of calomel, with 60 minims of laudanum, and an ounce of castor oil seven or eight hours afterwards, was in many cases sufficient to complete the cure. Other means are auxiliaries, but as such are useful, particularly the warm bath and stimulating frictions.

"In a disease, therefore, in which we have every reason to believe that venous congestion has taken place to a great extent, we conclude that the liver, from its peculiar circulation and structure, is more immediately liable to become seriously and permanently injured, it should not be omitted. We have before mentioned that Dr. James Johnson, of the Royal Navy, seems to have been the first to have pointed out the best method of cure; since most of the foregoing remarks were written, we have seen the second edition of that gentleman's valuable work, in which we find a strong corroborative testimony to the utility of blood-letting in this disease, or one somewhat similar to it, on the coast of Brazil, by Mr. Sheppard of Witney, without the assistance of any other remedy. The public are greatly indebted to Mr. Corbyn, of the Bengal Establishment, for his clear and comprehensive letter on this subject at a time when the disease was producing the most dreadful ravages: the early communication of his practice has been the means of saving thousands of lives in situations where Dr. Johnson's work might not be known." xlii.

The Board, in conclusion, are of opinion, that whatever other name be given to the disease, that of cholera should be discontinued. They agree with Sydenham in thinking, that from common cholera, both the disease they report on and that he described, are, *toto cœlo*, of a different nature. "Quisquis autem cholera morbi legitimi phænomena studiosè collegerit, fatebitur, morbum istum, quamvis eorundem symptomatum nonnullis stipatum, ab hoc nostro toto cœlo distare." Here is another dilemma in which the high cholera party are placed. They say that the present is an exclusively new disease, unlike that of Sydenham, who merely delineated common cholera. Yet Sydenham himself, no mean authority, like the Pythagorean, haud sordidus auctor naturæ verique, repudiates this dictum, and declares that what he saw was *not* common cholera. Sydenham, then describes a peculiar disease—the Indian Boards affirm that it is identical with the Indian cholera—the high cholera party assert that the Indian cholera is identical with our own epidemic—and yet they deny that our own epidemic is analogous with that described by Sydenham. This is merely one of the many and great inconsistencies of reasoning and argument into which certain persons have plunged themselves, by their breathless haste to prove the present epidemic a totally new and a most pestilential malady.

Here the general summary of the Board is terminated, and the individual reports, upon which it is chiefly founded, begin. These, as we said before, are forty in number. We shall throw the chief particulars into the form of a table, resembling that which we gave in our analysis of the Madras Report. Any features of particular interest or importance, will be mentioned in a succinct manner at the conclusion of the tabular statement.

TABULAR ANALYSIS of the INDIVIDUAL REPORTS transmitted to the BOMBAY PRESIDENCY.

| *Name of Medical Officer.* | *Premonitory Diarrhœa.* | *General Symptoms.* | *Consecutive Fever.* | *Contagious or not?* |
|---|---|---|---|---|
| 1. Assist. S. Robt. Wallace. *Seroor, July,* 1818. | none | purging, succeeded by vomiting, cramps—patients commonly cold in an hour. No blueness mentioned | none mentioned | no opinion given |
| 2. Dr. W. G. Burrell. *Seroor, July,* 1818. | none | at first languor, pyrexia in Europeans;—then vomiting & purging—cramps—sudden collapse | none mentioned | " cautious in reporting not infectious;" hospital attendants had it |
| 3. Assist. Surgeon Richards. *Punderpoor, July,* 1818. | none | spasms sometimes produced lock-jaw | none mentioned | no opinion |
| 4. Assist. S. Whyte, *Seroor, July,* 1818. | Watery purging from half-hour to 5 or 6. | usual symptoms. | whenever heat restored, out of danger. In a few, great heat afterwards, with bilious vomiting and purging | not contagious |
| 5. Assist. S. Daw. *Aurimgabad, July,* 1818. | none | sudden onset of usual symptoms—yellow eyes and sallow skin | none mentioned | " does not think it infectious—endemic best appellation" |
| 6. Mr. Craw. *Seroor July,* 1818. | none | lays great stress on spasm, and considers it in many Europeans a true tetanus | none mentioned | doubtful |
| 7. Assist. Surgeon Campbell. *Seroor.* | none | in Europeans pyrectic symptoms, followed by vomiting and purging | " not so soon convalescent as had been represented" | no opinion |
| 8. As. Surg. Tod. *Chumargoodee, Aug.* 1818. | none | disease peculiarly sudden—violent pain in bowels and extremities | none mentioned | no opinion |
| 9. As. S. Milward. *Ahmednuggur. Aug.* 1818. | none mentioned | nothing particular | none mentioned | ditto |

| *Name of Medical Officer.* | *Premonitory Diarrhœa.* | *General Symptoms.* | *Consecutive Fever.* | *Contagious or not?* |
|---|---|---|---|---|
| 10. Ass. S. Moyle. *Aug.* 1818. | none mentioned | thinks disease intimately connected with tetanus | none mentioned | no opinion |
| 11. Dr. Milne. *Poona, Aug.* 1818. | none mentioned. | ——— | ——— | ditto |
| 12. Assist. Surg. Wallace, *(iterùm.) Seroor, Aug.* 1818. | none mentioned | ——— | ——— | ditto |
| 13. Assist. Surgeon Richards, *(iterùm.) Punderpoor, Aug.* 1818. | none mentioned | ——— | ——— | ditto |
| 14. Assist. Surgeon White, *(iterùm.) Seroor, Aug.* 1818. | none mentioned | often begins by severe pain of abdomen—cramps of lower extremities—sometimes by sudden giddiness and confusion. | none mentioned | ditto |
| 15. Dr. Burrell, *(iterùm.) Seroor, Aug.* 1818. | none mentioned | cases of chronic dysentery aggravated during the epidemic—relapses not unfrequent | none mentioned | ditto |
| 16. Assist. Surg. White, *(iterùm.) Seroor, Aug.* 1818. | none mentioned | ——— | ——— | ditto |
| 17. Mr. Longdill. *Deccan.* | none mentioned | fatal when no pulse at wrist | ——— | ditto |
| 18. Mr. Gordon. *Satara, August.* | none mentioned | thinks brain affected before stomach and bowels—many became giddy in fields, fell, and died in a few minutes—jaw locked for a time in two cases | none mentioned | ditto |
| 19. Mr. Wallace, *(iterùm.) Seroor, August.* | none mentioned | ——— | none mentioned | ditto |
| 20. Mr. Wallace, *(iterùm.)* | " disease ushered in by purging" | ——— | ——— | gives many strong facts against contagion |
| 21. Mr. Robertson, *Keerky, August.* | none mentioned | very various—parts previously weakened generally much affected. | none mentioned | not contagious |

| *Names of Medical Officers.* | *Premonitory Diarrhœa.* | *General Symptoms.* | *Consecutive Fever.* | *Contagious or not?* |
|---|---|---|---|---|
| 22. Mr. Gordon, *(iterùm.) Satara, September* | none mentioned | ——— | ——— | ——— |
| 23. Assist. Surg. Henderson. *Kurrar, August.* | primary symptoms vary.—Generally watery stools first symptom. | hiccup in one fatal case | ——— | ——— |
| 24. Assist. Surg. Whyte, *(iterùm.)* | none mentioned | ——— | ——— | decidedly not |
| 25. Assist. Surg. Henderson, *(iter.)* | none ditto | ——— | ——— | not |
| 26. Mr. Surgeon Craw, *(iterùm.) Seroor, Aug.* | none ditto | ——— | ——— | ——— |
| 27. Capt. Sykes, *Punderpoor, Aug.* (Very inconsequential letter.) | in some cases diarrhœa at first. | ——— | ——— | contagious |
| 28. Mr. Whyte, *(iterùm.) Seroor, Aug.* | none mentioned | in bad cases no remedy beneficial in August purging first symptom—vomiting in from 2 to 8 hours | ——— | not contagious |
| 29. Assist. Surg. Anderson. *Poonah, Aug.* | none mentioned | great variation | none mentioned | " contagion scarcely merits notice" |
| 30. Mr. Surgeon Coats. | none | varieties | feverish reaction in every case that did well | " infectious under some very limited peculiarity of constitution" |
| 31. Mr. Surgeon Jukes. | none | pulse extremely various —sometimes headaches and giddiness;—lockjaw in one instance for some time. | none | not contagious, but very unlike common epidemic |
| 32. Dr. Taylor. | none | three different forms—1st, commences with slight pain in abdomen —2d, to this prostration rapidly succeeds—3d, patients fall down suddenly: very poorest classes suffered | none | seems to think it contagious, but says that only 3 out of 44 native assistants had it |

| *Names of Medical Officers.* | *Premonitory Diarrhœa.* | *General Symptoms.* | *Consecutive Fever.* | *Contagious or not ?* |
|---|---|---|---|---|
| 33. G. Olvey, Esq. | none | ———— | none | no decided opinion—no hospital attendants attacked |
| 34. F. Corbyn. *Centre Division, Grand Army.* | none | usual symptoms | none | "perfectly convinced it is not infectious."—No hospital attendants affected |

This concludes our tabular analysis of the individual reports. It will be seen on attentively referring to it, that although the reports are forty in number, the reporters are not so numerous, some gentlemen having forwarded to the central board, so many as two, three, or even more communications. Of the 22 reporters 10 give no opinion on the contagiousness or non-contagiousness of the disease. Now of those who give no opinion it is probable that the majority were non-contagionists, for this reason—that the contagionists were vastly in the minority throughout India, and they who entertain a general opinion are less likely to state it explicitly, than those who have found reasons for espousing peculiar and less commonly-received notions. Mr. Anderson, for instance, remarks that, " the idea of its (the cholera's) contagious nature is entertained by *so few*, and with so little reason, that it scarce merits notice."*

To pursue our comparison. Four are doubtful; one of the quartett, Dr. Taylor, appearing to incline against contagion, as he observes that none of the hospital attendants were attacked; whilst another, Dr. Burrell, leaning perhaps a little the other way, as he says he would be cautious in reporting the disease not infectious. Three are contagionists. Of these, one is non-professional, indeed unprofessional, namely, Captain Sykes. This gentleman, in answer to the observation that hospital attendants generally escaped, replies that he is convinced he would have had the disease, if he had not taken remedies to prevent it! He also feels certain that it could be proved to have been imported into every village, if persons were to set about it. This latter is a very judicious conclusion, and must have been dictated by a spirit, not altogether unprophetic. We know that, give a high-cholerist rope, and send him to Paris, he would prove to demonstration that the cook of Marshal Lobau was infected by M. Magendie, or the mate of a Calais steamer, who coming from Dover or the Thames, must, of course, have a pocket-full of cholera, to be disseminated from the said pouch or pocket throughout the circle of his acquaintance, or of all so unfortunate as to set foot upon his vessel.

To return. Ten are decidedly non-contagionists. The list then stands thus—no opinion, 10—doubtful, 4—contagionists, 3—non-contagionists, 10; 3 contagionists, in short, out of 22.

With respect to the premonitory diarrhœa, and the consecutive fever, the

* Report, p. 141.

table speaks for itself, and settles the question of *perfect identity* between cholera in England and cholera in Bombay. Yet what signify facts? We have no doubt, whatever, that persons will be found to persist in swearing to that identity, by asserting that diarrhœa and fever, as general rules, are no novel features; nay, more, that the Indian Reports will prove it.

Having disposed of these gentlemen thus, we redeem our promise of extracting a few of the more interesting circumstances from their reports. And first, of the mortality in Bombay. The following list comprises the amount of cases, of deaths under treatment, and of those ascertained by the police, where no treatment would seem to have been resorted to. The latter are probably below the actual numbers, and one-third or one-fourth may be added to them. The population of Bombay may amount to between 200 and 220 thousand, say 210,000. The number of ascertained cases 15,945, or $7\frac{1}{2}$ per cent.

"Abstract of Cases.

| 1818. | Cases. | Deaths. | Police. |
|---|---|---|---|
| August | 4400 | 256 | 409 |
| September | 4804 | 287 | 478 |
| October | 2411 | 146 | 181 |
| November | 824 | 44 | 29 |
| December | 806 | 64 | 72 |
| 1819. | | | |
| January | 889 | 114 | 125 |
| February | 517 | 27 | |
| | 14651 | 938 | 1294 |

Proportion of deaths in those cases where medicine was administered, 6.4 per cent."

The latter statement evinces the disproportion between the mortality of India and Europe. The following extract from the report of Mr. Whyte will display the influence of locality and of temperature, as well as the obscure connexion between the prevalence of cholera and fever.

"The Cholera Morbus made its first appearance here in the lines of the foot artillery, and attacked in succession every other corps, except the 4th light cavalry and 22d dragoons, which from situation or some other cause have hitherto escaped. I am the more inclined to think, that these corps owe their exemption to locality, from the following circumstance.—The Madras dooly bearers were encamped at the W. end of cantonment hill, between it and the nulla which separates the cavalry from the infantry lines; and while in that situation, several were attacked by the disease, and three or four died. At the request of the Assistant Surgeon of the 22d dragoons, whose duty it was to attend them, they were removed to a situation near the lines of that corps, that he might be at hand to afford them the most speedy aid; and since their removal, I understand no fresh cases of cholera have occurred. Since the epidemic showed itself here, the days have been exceedingly close and sultry, and about sunset a piercing cold wind has set in from the S. W.: this wind blows down the channel of the nulla mentioned above, turns the corner of cantonment hill, and blows over the cantonments. From want of a thermometer, I could not judge accurately of the degree of heat; but from my sensations, I think its range must have been extensive. The class of people which has principally suffered from this disease, is composed of the poor, badly clothed, or fed, and those of a debilitated constitution ; or else of men, in whom the perspiratory process has been highly excited during the day, whether by violent exercise or exposure to the sun ; and in this way sometimes the stoutest and healthiest men suffer. While this disease bears all the characters of an epidemic, undoubtedly depending upon some peculiar and unknown properties in the atmosphere, it appears to be more readily caused by currents of that atmosphere blowing from particular quarters ; and what is of great practical importance, a situation sheltered from these currents, a situation for example, in the lee of a hill, is particularly free from the influence of this epidemic. It appears to me that, in some constitutions, this cold wind, instead of producing Cholera, causes a regular attack of ague and fever. You will observe by the abstract of the 7th, that 18 cases of fever were admitted last week, although the period of their admission was not the springs, when that disease commonly shews itself: in few of these men did more than one fit occur. The above impression was made stronger on my mind, from what took place in my own person. After all my perspiratory pores had been kept open some time, in a crowded hospital, on going across the parade, I was suddenly seized with a cold shivering and trembling fit, which lasted some time after my return home. All my thoughts were fixed on Cholera. By means of the pediluvium and mulled port wine, however, I restored warmth and comfortable feelings, but suffered a smart febrile attack after going to bed, which kept me hot and restless during the night ; but from which I, in the morning, arose free though languid. I think, that had my constitution been so predisposed, the same cause which produced fever, would have brought on cholera morbus." 13.

The same gentleman offers us an instance of that rather rara avis, a spontaneous cure, or something like it.

" I at first thought, that in a disease of so much violence, there was no such thing as a spontaneous cure ; but a man who had been affected three days, was brought to the hospital this morning, in whom the spasms had almost entirely gone off, and whose pulse could be felt at the wrist, although from the description he must have sustained a violent attack, and he had taken no medicine." 20.

Mr. Daw relates a curious instance of the escape of one body of men, probably from prudential conduct, whilst another body, less cautious, suffered severely.

" Two bodies of men, one of nearly three hundred, and the other about one hundred (this is very nearly the numbers but not exact) were in adjoining situations, when the disease broke out in the place where these troops were. The 100 men immediately determined, that, by great temperance and care, and by not exposing themselves unnecessarily, and in particular by avoiding the night air, to endeavour to escape the disease. It succeeded so well that only one man had an attack of it ; while the other body of men, the 300 who took no such precaution, lost one-tenth of their whole number. You may think from the manner in which I have written this, that I have no distinct information as to numbers ; but had I time, I could easily procure the exact statement, which I am quite certain would very nearly agree with what I have said from memory." 31.

Such has been the capriciousness of cholera that one or two instances of this kind are worth little, at least as foundations for reasoning. During the prevalence of cholera over the small island of Salsett, some villages escaped. Two months after the disease had disappeared from the island, it broke out in one of those same villages, and destroyed 23 out of a population of 80. Had quarantine been adopted in the first instance, to it the original escape, and to its infraction the subsequent desolation would no doubt have been attributed with some plausibility by the post-hoc-ergo reasoners.

Mr. Wallace, in his report, notices the general prevalence either of febrile symptoms, or bowel-complaint among all persons, during the existence of cholera. How much this has been the case in London, those who have any practice can testify. In this country some practitioners have been astonished by comparatively sudden deaths of patients apparently convalescent. Several such cases are mentioned in these reports. Two such examples, but not strong ones, are given by Mr. Whyte.

"The European lived three days or more from the attack of the disease; warmth, a natural pulse, perspiration, and yellow-coloured stools, had returned; but a degree of coma came on, for which he was bled; and I believe he died about the third day, coldness having re-appeared. The sepoy was attacked at day-light, admitted at 11 o'clock, and died at 4 the following evening. I saw him a few hours before death, and could just perceive his pulse; vomiting was completely suppressed, and purging nearly so, although tenesmus urged him to remain almost continually upon the stool. However, I thought him in a fair way to recover, and was astonished a few hours after to receive a report of his death." 65.

The following circumstances strongly militating against contagion, are related by the same gentleman.

"Convinced, as I am, of the total absence of contagion in this disease, I have observed the late revival in some measure of this opinion with some degree of pain. Surely, if it was at all contagious, the fact of its being so could not long remain doubtful. In the general hospital here, there were three sepoys, who resided continually from the first appearance of the epidemic, inhaling at every inspiration by day and night, mouthfuls of infection. If the atmosphere was really loaded with contagious effluvia, arising from the bodies of the numerous inhabitants of the hospital, the escape of these men (which has been complete) would be miraculous indeed, living as they were in the very midst of these effluvia, and so near their source. Allowing that the constant habit of doing so procured them an exemption from the influence of this contagion, the same thing cannot be said of the friends and relations who were attending upon the patients, nor of six dooly bearers, changed daily, and who used to assist the sick into and out the bath, and in every other way; thereby exposed to be infected with the disease, whether it is conveyed through the medium of the atmosphere, or by touch: and yet I have not known one instance of dooly bearers, friends, and attendants of the sick being so infected; nor have any of our hallalchores, or hospital assistants suffered. One of our correspondents supposes, that the disease has travelled in a direct route at the easy rate of 15 miles a day, and *believes, if it could be proved,* that it has not shewn itself in any village, that had remained insulated or unconnected with other villages, where the disease was. Until this *is proved,* it is quite as easy to believe the contrary. In the mean time, we have seen it affecting a particular part in one cantonment for days, without reaching another part, although a constant communication was kept up between these parts all the while." 105.

But we dare not proceed farther. With respect to the treatment, we can only say that, on the whole, the testimony of the reporters greatly preponderates in favour of bleeding and calomel. On the other items it is unnecessary for us to say any thing at present. It is singular that throughout these reports no mention is made of blueness, that favourite colour of the cholerics of Europe. Here we conclude the Bombay Report. It was drawn up suddenly after the irruption of the epidemic, and is much more incomplete than either of the other two reports from Bengal and Madras. Nevertheless it is worthy of record, and we give it a place for the purpose of future reference, when the original document will not be procurable.

